# Is tattooing associated with an increased risk of cancer? A systematic review and meta-analysis

**DOI:** 10.1007/s12094-026-04388-4

**Published:** 2026-06-06

**Authors:** Gabriel Caruso Novaes Tudella, Maria Luiza Rodrigues Defante, Pedro Sandes Pereira, Giovana Menegucci, Augusto Utida Klostermann, João Vitor Andrade Fernandes, Eduarda Lima Ornelas Pinto, Ocílio Ribeiro Gonçalves, António Balele Kanda João, Lília Maria Lima de Oliveira

**Affiliations:** 1 Department of Medicine, Federal University of Santa Maria, Santa Maria, Brazil; 2 Department of Medicine, Redentor University Center, Itaperuna, Brazil; 3 Department of Medicine, Bahiana School of Medicine and Public Health, Salvador, Brazil; 4 Medical Sciences Center, Federal University of Paraíba, João Pessoa, Brazil; 5 Department of Medicine, Centro Universitário de Belo Horizonte (UniBH), Belo Horizonte, Brazil; 6 Department of Medicine, Federal University of Piauí, Teresina, Brazil; 7 Institute of Health Sciences, Agostinho Neto University, Luanda, Angola; 8 Principles and Practice of Clinical Research (PPCR), Postgraduate Program, Harvard T. H. Chan School of Public Health, Boston, MA USA

**Keywords:** Tattooing, Neoplasm, Lymphoma, Melanoma, Meta-analysis

## Abstract

**Background:**

Tattoo inks may contain carcinogenic compounds, and pigment migration to lymphatic tissues raises concerns regarding potential cancer risk. Epidemiological evidence remains inconclusive.

**Objectives:**

To assess the association between tattoo exposure and the incidence of skin cancer and hematological malignancies.

**Methods:**

PubMed, Embase, and the Cochrane Library were searched from inception to January 2026 for cohort and case–control studies comparing cancer incidence in tattooed versus non-tattooed adults. Random-effects meta-analyses using restricted maximum-likelihood estimators were performed to pool odds ratios (ORs) with 95% confidence intervals (CIs). Heterogeneity was quantified using *I*^2^ statistics. Risk of bias was assessed using ROBINS-E, and certainty of evidence was evaluated with GRADE. Leave-one-out sensitivity analyses were conducted.

**Results:**

Seven observational studies including 140,841 participants were analyzed. Tattoo exposure was not associated with overall skin cancer (OR 0.92; 95%CI 0.83–1.04; *I*^2^ = 0%; *p* = 0.179). Stratified analyses by number of tattoo sessions showed no significant associations: one session (OR 1.08; 95%CI 0.55–2.12; *I*^2^ = 92.4%), two-to-three sessions (OR 0.90; 95%CI 0.62–1.32; *I*^2^ = 63.2%), and ≥ 4 sessions (OR 0.70; 95%CI 0.29–1.70; *I*^2^ = 88.5%). For hematological malignancies, pooled analysis showed no significant association (OR 1.02; 95%CI 0.78–1.32; *I*^2^ = 64.9%; *p* = 0.910). Subtype analyses were non-significant: non-Hodgkin lymphoma (OR 1.00; 95%CI 0.73–1.36; *I*^2^ = 3.7%), Hodgkin lymphoma (OR 1.19; 95%CI 0.59–2.40; *I*^2^ = 91.7%), diffuse large B-cell lymphoma (OR 0.90; 95%CI 0.57–1.44; *I*^2^ = 61.7%), T-cell lymphoma (OR 1.07; 95%CI 0.61–1.88; *I*^2^ = 0%), and follicular lymphoma (OR 0.99; 95%CI 0.72–1.36; *I*^2^ = 1.9%). Sensitivity analysis excluding one influential study rendered the association for overall hematological malignancies statistically significant (OR 1.20; 95%CI 1.05–1.39; *I*^2^ = 0%). Certainty of evidence ranged from low to moderate.

**Conclusions:**

Tattoo exposure was not associated with increased skin cancer risk and showed no significant association with hematological malignancies in primary analyses. A significant association emerged only after sensitivity analysis, warranting cautious interpretation and further prospective investigation.

**Supplementary Information:**

The online version contains supplementary material available at https://doi.org/10.1007/s12094-026-04388-4.

## Introduction

The prevalence of tattoos and permanent makeup (PMU) is estimated at 10–20% in the European population, while in the United States, approximately 31% of individuals report having at least one tattoo [1–3]. In addition, tattooing has also expanded rapidly among adolescents and young adults. However, this widespread practice has raised growing toxicological and public health concerns, since tattoo inks contain variable levels of substances classified as carcinogenic or probably carcinogenic by the International Agency for Research on Cancer, including polycyclic aromatic hydrocarbons (PAHs) and primary aromatic amines (PAAs) [7–10]. Considering that, the composition of these inks recently came under scrutiny, leading the European Chemicals Agency (ECHA) to restrict chemicals found in tattoo inks [11]. These concerns culminated in 2022, when the European Union began regulating tattoo inks to mitigate potential health risks [12].

There is increasing evidence that tattoo pigments do not remain confined to the site of injection but can migrate to regional and distant lymph nodes, such as seeing on histopathological studies that have shown that pigment particles can be transported through lymphatic and blood vessels by migrating immune cells [13–17]. Additionally, dermal pigment concentrations gradually decline during wound healing and continue to decrease over the years, reinforcing the existence of ongoing degradation and translocation processes [18]. In this context, the detection of pigment particles and metal-based compounds in distant lymphatic tissues suggests that ink constituents may interact with the lymphoid systems. Beyond pigment migration, tattooing may provoke chronic inflammatory and immune responses that persist for years [19, 20]. These immune effects are relevant to carcinogenic potential, particularly regarding melanoma malignancies, which exhibit high mutational burdens and complex immune microenvironments [21].

Two previous systematic reviews and one meta-analysis found no statistically significant evidence of an association between tattoo exposure and hematological malignancies [22–24]. However, a more comprehensive and in-depth evaluation of the potential association between tattooing and the risk of both skin cancer and hematological malignancies is warranted, as isolated studies suggesting links with cutaneous neoplasms have not yet been systematically synthesized. In addition, the previous reviews performed a quantitative synthesis of effect estimates with a limited evidence base. Therefore, this meta-analysis aimed to evaluate the existing evidence and strengthen the statistical assessment on the relationship between tattoo exposure and the incidence of skin cancer and hematological malignancies.

## Materials and methods

### Protocol and registration

This systematic review and meta-analysis followed Preferred Reporting Items for Systematic Reviews and Meta-Analyses (PRISMA) [25], Cochrane Handbook for Systematic Reviews of Interventions [26], and Meta-analysis of Observational Studies in Epidemiology (MOOSE) [27] guidelines (Tables S1, S2), and was prospectively registered in PROSPERO (CRD420251076015). Detailed methodological procedures are described in the Supplementary Material.

### Eligibility criteria

We included observational studies with a cohort or case–control design that enrolled adults aged ≥ 18 years. The population of interest comprised individuals with tattoos (exposure group) compared with individuals without tattoos (control group). Eligible studies were required to report incident cases of skin cancer and/or lymphoma as outcomes. No restrictions were applied regarding setting or length of follow-up. We excluded reviews, case reports, case series, conference abstracts, protocols, studies with overlapping populations, and studies with insufficient data—such as those that did not report any proportion (%) or odds ratios (ORs) with 95% confidence intervals (CIs) for outcomes of interest.

### Search strategy, study selection, and data extraction

We searched PubMed, Embase, and the Cochrane Library from inception to January 2026, with no language restrictions. Reference lists of eligible papers and relevant reviews were screened manually to identify additional studies. Two reviewers (G.C.N.T. and G.M.) independently screened titles and abstracts, assessed full texts for eligibility, and extracted data using a predefined template. Extracted variables included study design, sample size, age, sex, participant demographics, inclusion criteria, data collection method, tattoo size, number of sessions, and confounding variables—such as smoking habit. During data extraction, overall lymphoma was defined as a composite outcome aggregating all lymphoma subtypes (e.g., HL and NHL), and was analyzed separately from multiple myeloma, which was not included in the pooled lymphoma estimates. Discrepancies were resolved by discussion with two additional reviewers (A.U.K. and P.S.). The complete search algorithms, inclusion flow, and extraction domains are provided in Supplementary Material (eMethods 1, 2).

### Outcomes’ definition

The outcomes of interest encompassed both cutaneous and hematological malignancies, with definitions grounded in registry-verified diagnoses and standardized classification systems. Skin cancer included melanoma (ICD-10: C43) and nonmelanoma skin cancer (ICD-10: C44), with basal cell carcinoma excluded in the Danish Twin Tattoo Survey to preserve the specificity of case–cotwin analyses. Hematological outcomes encompassed malignant lymphomas, with subtypes classified according to ICD-O-3 and categorized into HL, NHL including diffuse large B-cell lymphoma, follicular lymphoma, chronic lymphocytic leukemia/small lymphocytic lymphoma, T-cell lymphomas, and other B-cell variants.

All outcomes represented incident, first-time diagnoses. Diagnoses were confirmed via national or regional cancer registries, ensuring morphological verification and high coverage. Analyses were stratified by tattoo exposure frequency and cancer subtype, with sensitivity assessments conducted to evaluate the influence of individual studies on pooled estimates. Additionally, to ensure consistency across study designs, data from both case–control and cohort studies were harmonized by reconstructing standardized 2 × 2 contingency tables based on exposure (tattoo vs. no tattoo) and outcome (cancer vs. no cancer). This approach enabled the derivation of comparable effect estimates across studies while preserving the interpretability of the original data structures.

### Quality assessment and assessment of clinical relevance

Two reviewers independently (G.C.N.T. and G.M.) assessed the risk of bias in observational studies using the ROBINS-E [28] (Risk of Bias in Non-randomized Studies of Exposure) tool, summarized with the ROBVIS platform (eMethods 3). The certainty of evidence was appraised using the GRADE (Grading of Recommendations Assessment, Development, and Evaluation) framework, classifying the overall confidence as high, moderate, low, or very low based on risk of bias, inconsistency, indirectness, and imprecision. Any disagreement between reviewers was resolved by discussion with third and fourth authors (P.S. and A.U.K.).

### Statistical analysis

The primary outcome was the incidence of skin cancer or lymphoma among tattooed individuals compared with non-tattoo (controls), expressed as ORs with 95% CIs. Random-effects meta-analyses were performed using restricted maximum-likelihood (REML) estimators. Heterogeneity was assessed with the Q statistic and quantified with the *I*^2^ statistic, with *p* < 0.05 considered statistically significant and *I*^2^ values interpreted as follows: < 40% (low), 40–60% (moderate), and > 60% (substantial). Due to the limited number of included studies, fewer than 10 per analysis, it was not feasible to conduct Egger’s regression test to assess publication bias or perform a meta-regression. Leave-one-out sensitivity analyses were performed, sequentially excluding each study to assess its influence on pooled results. All *p* values were two-sided for meta-analyses (*α* = 0.05). Analyses were conducted in R version 4.5.0 (R Foundation for Statistical Computing, Vienna, Austria). A full description of statistics is available in Supplementary Material (eMethods 4).

## Results

### Study selection and characteristics

**Fig. 1:**
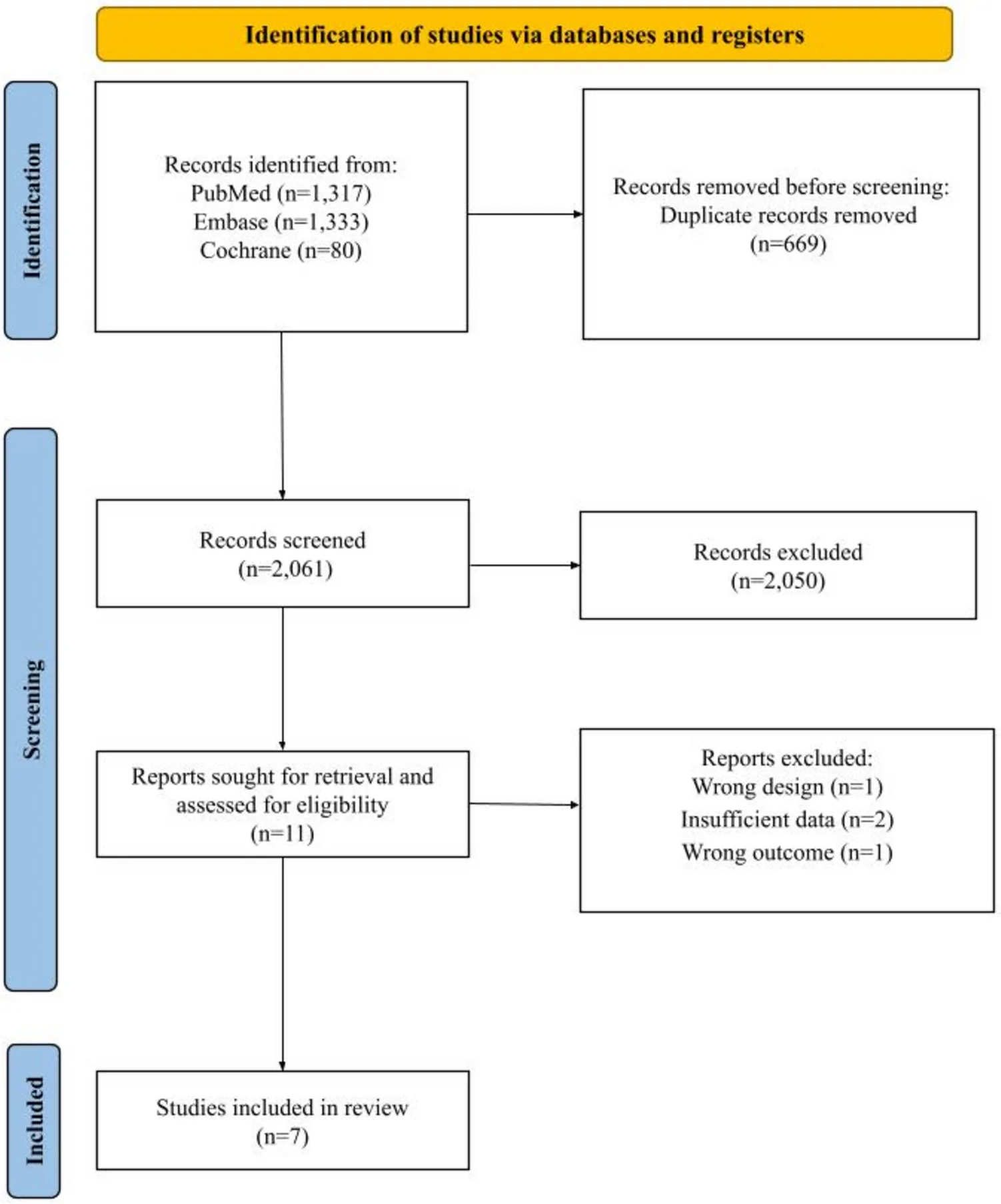
PRISMA 2020 flow diagram

The initial search yielded 1317 studies from PubMed, 1333 studies from Embase, and 80 studies from Cochrane. After the removal of 669 duplicates, 2061 records underwent title and abstract screening, of which 2050 were excluded. A total of 11 reports were assessed for full-text eligibility; among these, four were excluded due to wrong study design (*n* = 1), insufficient data (*n* = 2), or wrong outcome (*n* = 1). Ultimately, seven studies were included in the final analysis [8, 29–34]. The PRISMA flow diagram is shown in Fig. 1, and the supplementary material (eMethods 1) provides a detailed account of the reasons for exclusion following full-text assessment, as well as additional details of the included studies.

### Characteristics of included studies

A total of seven studies were included in the baseline characterization, comprising 140,841 participants overall. Across the included articles, study populations encompassed individuals diagnosed with lymphoma, melanoma, nonmelanoma skin cancer, and other hematological malignancies, as well as matched control groups drawn from the general population. Regarding the geographical distribution, most studies were conducted in high-income countries, with Sweden and the United States each contributing two studies, followed by Denmark, France, and Canada with one study each. Six studies reported sex distribution. 61,850 (46.1%) were male and 72,336 (53.9%) were female. The included studies collected data via surveys, national registers, and telephone interviews, using matched or population-based controls. Among studies that reported age at first tattoo, the age ranged from 22 to 28 years. All studies applied adjustments for key confounders—including age, sex, education, smoking, BMI, UV exposure, and family history—though methods varied across studies. Detailed baseline characteristics of each study are presented in Tables 1 and 2.

**Table 1:** Baseline characteristics of included studies

A. Case–control studies									
Study (year)	Study design	Country	Groups	Sample size (*n*)	Male/female	Age (years)	Exposure assessment	Outcome ascertainment	Adjusted confounders
Clemmensen (2025) [29]	Case–control (co-twin)	Denmark	Cases (lymphoma)	32	21/11	50 (44–53)	Self-reported tattoo status, size, color	Danish Cancer Registry	Age (timescale), sex stratification; IPW
			Controls	34	15/19	42 (30–50)			
			Cases (skin cancer)	119	46/73	50 (46–54)			
			Controls	104	42/62	42 (30–50)			
Liljedahl (2025) [32]	Case–control (population-based)	Sweden	Cases (cSCC)	1600	653/947	56	Self-reported tattoo history	National registry	UV index, phenotypic index, education, income, smoking
			Controls	4551	1756/2795	56			
McCarty (2025) [33]	Case–control (population-based)	USA	Cases (melanoma)	641	361/280	NA	Self-report (BRFSS)	Cancer registry	Age, sex, race, education, BMI, smoking, physical activity (PSM)
			Controls	8200	4340/3860	NA			
McCarty (2024) [8]	Case–control (population-based)	USA	Cases (hematologic)	820	NA	NA	Self-report (BRFSS)	Cancer registry	Age, sex, race, education, smoking, prior cancer
			Controls	5835	NA	NA			
Nielsen (2024) [30]	Case–control (population-based)	Sweden	Cases (lymphoma)	1392	769/623	NA	Self-reported questionnaire	Cancer registry	Education, income, smoking, marital status (DAG-informed)
			Controls	4179	2219/1960	NA			
Warner (2020) [31]	Case–control (population-based)	Canada	Cases (NHL)	737	431/306	NA	Self-reported questionnaire	BC Cancer Registry	Age, sex, ethnicity, education, BMI, family history
			Controls	781	421/360	NA			
			Cases (MM)	373	223/150	NA			
			Controls	369	211/158	NA			

B. Cohort studies									
Study (year)	Study design	Country	Exposure group	Sample size (*n*)	Male/female	Age (years)	Exposure assessment	Outcome ascertainment	Adjusted confounders
Mo (2025) [34]	Population-based cohort	France	Tattooed	12,669	4736/7933	40 (32–49)	CONSTANCES questionnaire (self-report)	National registry	Age, sex, education, smoking, BMI, income, skin type, tanning intensity, sunburns, healthcare access
			Non-tattooed	98,405	45,606/52,799	51 (40–61)			

*cSCC* cutaneous squamous cell carcinoma, *NHL* non-Hodgkin lymphoma, *MM* multiple myeloma, *IPW* inverse probability weighting, *PSM* propensity score matching, *DAG* directed acyclic graph, *BRFSS* Behavioral Risk Factor Surveillance System

**Table 2:** Baseline characteristics of tattoo exposure in the study population

A. Case–control studies							
Study (year)	Study design	Groups	*N* total	Tattooed individuals, *n* (%)	Number of sessions, *n* (%)^a^	Tattoo color, *n* (%)^b^	Age at first tattoo, median (IQR)
Clemmensen (2025) [29]	Case–control (twins)	Cases (lymphoma)	32	10 (31%)	NR	Black: 9; red: 6	28 (26–36)
		Controls	34	11 (32%)	NR	Black: 10; red: 6	22 (18–27)
		Cases (skin cancer)	119	30 (25%)	NR	Black: 29; red: 11	24 (18–32)
		Controls	104	25 (24%)	NR	Black: 24; red: 9	24 (18–37)
Liljedahl (2025) [32]	Case–control (population-based)	Cases (cSCC*)	1600	236 (15%)	1: 42%; 2–3: 37%; ≥ 4: 21%	B/G: 34%; B/G + color: 45%; color only: 20%^b^	30 (NR)
		Controls	4551	802 (18%)	1: 48%; 2–3: 33%; ≥ 4: 19%	B/G: 34%; B/G + color: 49%; color only: 17%^b^	30 (NR)
McCarty (2024) [33]		Cases (lymphoid)	641	11% overall (22% in Hodgkin lymphoma)	NR	NR	NR
	Case–control (population-based)	Cases (myeloid)	179	29 (16%)	NR	NR	NR
		Controls	8200	1230 (15%)	NR	NR	NR
McCarty (2025) [8]	Case–control (population-based)	Cases (melanoma)	1167	140 (12%)	1: 56%; 2–3: 26%; ≥ 4: 17%	NR	< 20y: 31%; ≥ 20y: 69%
		Controls	5835	855 (15%)	1: 33%; 2–3: 32%; ≥ 4: 35%	NR	< 20y: 37%; ≥ 20y: 62%
Nielsen (2024) [30]	Case–control (Population-based)	Cases (lymphoma)	1398	289 (21%)	1: 43%; 2–3: 32%; ≥ 4: 23%	B/G: 39%; B/G + color: 46%; color only: 15%^b^	23 (19–35)
		Controls	4193	735 (18%)	1: 42%; 2–3: 29%; ≥ 4: 28%	B/G: 35%; B/G + color: 49%; color only: 16%^b^	22 (18–35)
Warner (2020) [31]	Case–control (population-based)	Cases (NHL)	737	42 (6%)	NR	NR	< 35y: 36; ≥ 35y: 6
		Controls	781	46 (6%)	NR	NR	< 35y: 35; ≥ 35y: 11
		Cases (MM)	373	37 (10%)	NR	NR	< 35y: 19; ≥ 35y: 12
		Controls	369	35 (10%)	NR	NR	< 35y: 12; ≥ 35y: 18

B. Cohort studies							
Study (year)	Study design	Exposure group	*N* total in group	Cancer events, *n* (%)	Number of sessions, *n* (%)^a^	Tattoo color, *n* (%)^a^	Age at first tattoo, median (IQR)
Mo (2025) [34]	Cohort (retrospective)	Tattooed	12,669	87 (0.7%)	NR	NR	NR
		Non-tattooed	98,405	1,702 (1.8%)	NR	NR	NR

*B/G* black and gray, *cSCC* cutaneous squamous cell carcinoma, *MM* multiple myeloma, *NHL* non-Hodgkin lymphoma, *NR* not reported.

^a^Percentages for number of sessions and tattoo color represent the distribution within the tattooed group

^b^Exact number not reported.

In case–control studies, percentages represent the proportion of tattooed individuals within each group (cases or controls), using group-specific denominators. In cohort studies, percentages represent the proportion of cancer events within each exposure group.

### Pooled analysis of included studies

**Fig. 2:**
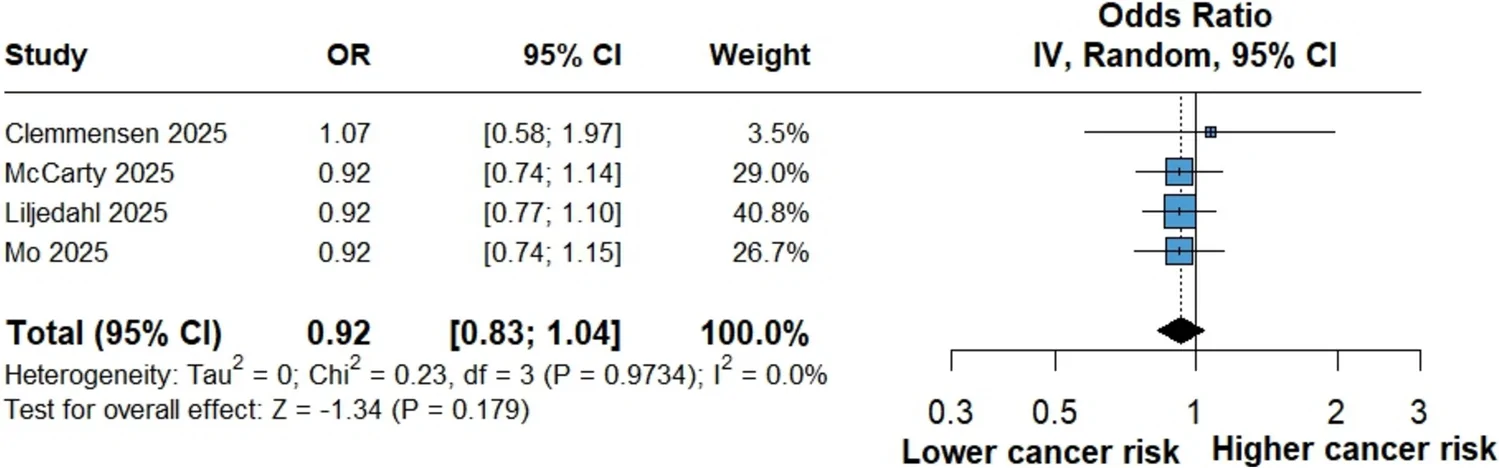
Odds ratio of the risk of skin cancer among patients ever tattooed. *CI* confidence interval, *OR* odds ratio, *IV* inverse variance

Across four studies evaluating skin cancer, tattoo exposure was not significantly associated with overall risk (OR 0.92; 95%CI 0.83–1.04; *I*^2^ = 0%; *p* = 0.179; Fig. 2; Table S5). Stratified analyses according to number of tattoo sessions likewise demonstrated no significant associations: one session (OR 1.08; 95%CI 0.55–2.12; *I*^2^ = 92.4%), two-to-three sessions (OR 0.90; 95%CI 0.62–1.32; *I*^2^ = 63.2%), and four or more sessions (OR 0.70; 95%CI 0.29–1.70; *I*^2^ = 88.5%) (Figures S2, S3).

**Fig. 3:**
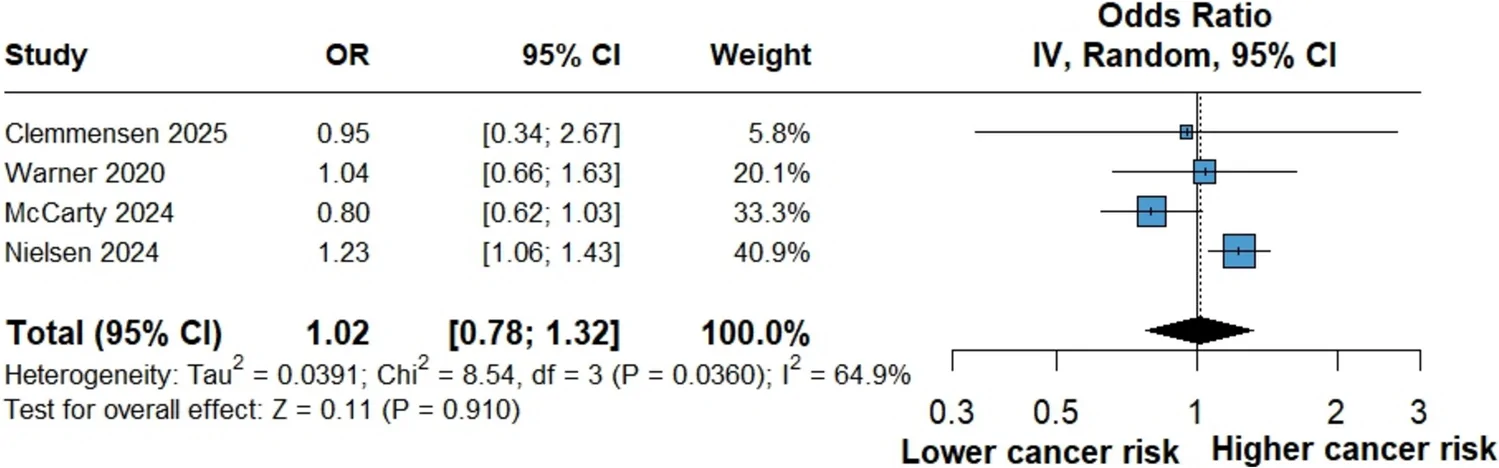
Odds ratio of risk of any lymphoma among patients tattooed. *CI* Confidence interval, *OR* odds ratio, *IV* inverse variance

For hematological malignancies, four studies contributed to the pooled estimate. No significant association was observed for overall lymphoma (OR 1.02; 95%CI 0.78–1.32; *I*^2^ = 64.9%; *p* = 0.910; Fig. 3; Table S6). Subtype analyses were similarly non-significant, including non-Hodgkin lymphoma (OR 1.00; 95%CI 0.73–1.36; *I*^2^ = 3.7%), Hodgkin lymphoma (OR 1.19; 95%CI 0.59–2.40; *I*^2^ = 91.7%), diffuse large B-cell lymphoma (OR 0.90; 95%CI 0.57–1.44; *I*^2^ = 61.7%), T-cell lymphoma (OR 1.07; 95%CI 0.61–1.88; *I*^2^ = 0%), follicular lymphoma (OR 0.99; 95%CI 0.72–1.36; *I*^2^ = 1.9%), and other B-cell subtypes (OR 1.14; 95%CI 0.83–1.55; *I*^2^ = 0%) (Figures S4, S5, S6, S7, S8, S9).

### Sensitivity analysis

Leave-one-out analyses for skin cancer demonstrated stable pooled estimates with persistently absent heterogeneity (*I*^2^ = 0%) (Figure S10). For overall lymphoma, exclusion of McCarty et al. (2024) yielded a statistically significant association (OR 1.20; 95%CI 1.05–1.39; *I*^2^ = 0%; Figure S11). For non-Hodgkin lymphoma, pooled ORs ranged from 0.79 to 1.11 across sequential exclusions, and removal of Nielsen et al. (2024) or McCarty et al. (2024) eliminated residual heterogeneity (*I*^2^ = 0%; Figure S12). In diffuse large B-cell lymphoma, effect estimates ranged from 0.67 to 1.06, with heterogeneity resolving after exclusion of Nielsen et al. (2024) (*I*^2^ = 0%; Figure S13).

### Risk of bias and certainty of evidence

**Fig. 4:**
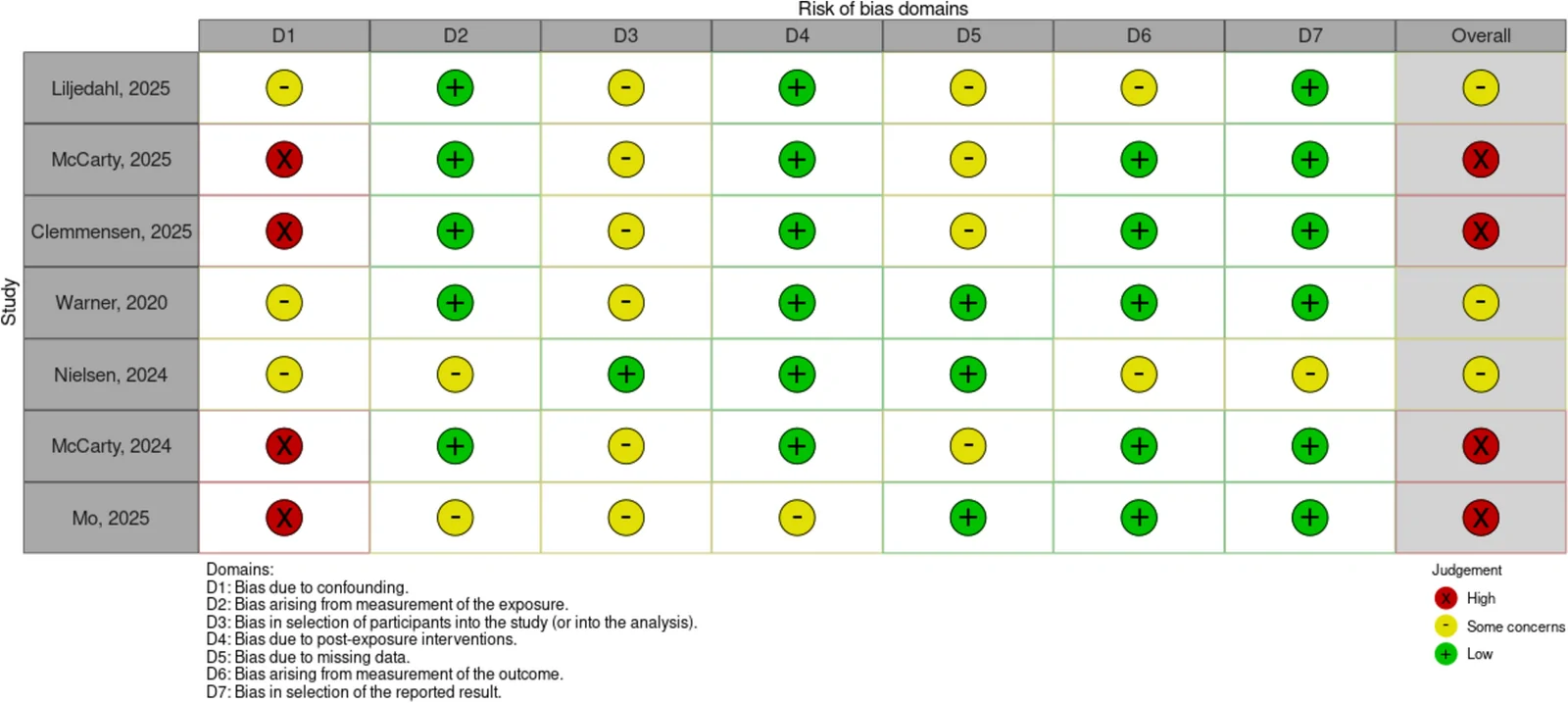
Risk-of-bias assessment of the included studies was conducted using the Risk of Bias in Non-randomized Studies of Exposures tool (ROBINS-E)

According to ROBINS-E, four of seven studies were judged at high overall risk of bias, while three were rated as having some concerns; none were considered low risk. Confounding was the predominant source of bias, frequently insufficiently controlled. Exposure classification also raised concerns in most studies. Other domains, including participant selection, deviations from intended exposure, missing data, outcome measurement, and selective reporting, were generally assessed as low risk (Fig. 4; Table S3).

Using GRADE, certainty of evidence for skin cancer was low, driven by observational design, serious risk of bias, and imprecision. Certainty for analyses stratified by number of tattoo sessions ranged from low to very low. For overall hematological malignancies, certainty was moderate, whereas for Hodgkin lymphoma, non-Hodgkin lymphoma, and specific subtypes it was low, primarily due to risk of bias and limited sample size (Table S7).

## Discussion

In this meta-analysis of seven observational studies comprising 140,841 participants, we evaluated the association between tattoo exposure and the risk of skin cancer and hematologic malignancies. A statistically significant pooled association was observed for overall hematologic malignancies in sensitivity analysis, whereas no significant associations were identified for skin cancer and most lymphoma subtypes. Although significant in sensitivity analysis, this finding should be interpreted cautiously, as it is exploratory and does not alter the primary null result. Given the observational design of the included studies, these findings should be interpreted as associative rather than causal. Although most studies adjusted for confounding using varied methodological approaches, residual confounding, exposure misclassification, and selection bias cannot be fully excluded.

Tattoos have become increasingly common among young adults [35]. This growing acceptance has been accompanied by a rising number of reported tattoo-related dermatoses and skin cancers. Case reports describing melanoma, basal cell carcinoma, and squamous cell carcinoma (SCC) arising within tattooed skin have multiplied, although a definitive epidemiological link remains unresolved [35–37]. Biologically, tattooing introduces variable and often poorly regulated mixtures of metallic salts and organic dyes directly into the dermis [38]. These exogenous products might be genotoxic, cytotoxic, or generate reactive oxygen species under light exposure [9]. These factors raise concerns about potential oncogenic mechanisms, despite the current lack of clear causal evidence.

Biologically plausible hypotheses are consistent with findings from previous systematic reviews describing a gradual accumulation of tattoo-associated cutaneous tumors over time [24, 39]. These observations lend mechanistic coherence and suggest partially supportive signals, particularly for keratinocytic neoplasms reported in selected cases [24, 40]. However, the absolute number of documented tumors remains small when contrasted with the widespread and increasing prevalence of tattooing, and the observed patterns may be substantially influenced by diagnostic confounders, including lesion masking, delayed detection, and surveillance bias [41, 42]. Consistent with the findings of our meta-analysis, robust epidemiological evidence to support a causal association between tattoo exposure and skin cancer is currently lacking.

The observation that patients who develop skin cancer within tattooed areas tend to be younger and exhibit a shorter latency period between tattoo application and tumor diagnosis may reflect both demographic patterns and evolving toxicological factors [9]. As more young people acquire multiple tattoos the probability that a coincidental malignancy arises within tattooed skin naturally increases [9]. An additional explanation relates to changes in the chemical makeup of tattoo inks. Over the past two decades, pigment formulations have shifted: traditional inorganic pigments containing metals, such as mercury, cadmium, and cobalt, are now less common, yet modern red and black inks still frequently incorporate potentially hazardous substances, including azo pigments and polycyclic structures, several of which—or their photodegradation or laser-induced byproducts—are classified as carcinogenic or potentially carcinogenic [12]. Despite the large overall sample size, the number of cancer events within exposed subgroups was relatively small, particularly for specific malignancy subtypes, which may have limited statistical power to detect modest associations.

Studies investigating the association between tattoo exposure and cancer face substantial challenges in adequately controlling for confounding, because of the complex interplay between lifestyle, environmental, and genetic factors. Although twin cohorts that inherently account for shared genetic and early life environmental influences, and population-based case–control studies adjusting for age, sex, ethnicity, and smoking represent methodological advances, residual confounding remains a major concern [29]. The lack of information on sun exposure in that study is a limitation. UV exposure is particularly problematic, as it is a well-established risk factor for cutaneous SCC and melanoma, and individuals with tattoos may engage in behaviors that either increase sun exposure or increase sun protection [32]. The Utah study, for example, found that tattooed melanoma cases were more likely to use tanning beds and report sunburns, suggesting that unmeasured UV-related behaviors may inflate observed risks [8]. Specifically, tattoos also interact with UV-driven photodegradation of pigments into potentially toxic compounds. These elements underscore the difficulty of disentangling the effects of tattoo pigments from broader environmental and behavioral cofactors, complicating causal interpretation in epidemiologic research.

The relationship between tattoos and the timing of both cutaneous and hematological malignancies shows substantial variability, making it difficult to define a single latency period or establish straightforward causality [29]. In skin cancers reported primarily through case reports, which inherently limit causal inference, latency ranged widely: SCCs occurred from 10 days to 50 years after tattooing, and keratoacanthomas, often viewed as low-grade SCC variants, appeared between 3 weeks and 9 years, frequently within the first months, likely reflecting trauma-induced processes [9, 36, 43, 44]. For hematological malignancies, epidemiologic data indicate that long latencies are also plausible; in the Swedish cohort, lymphoma risk increased both within 2 years after tattooing and again after 11 or more years, mirroring patterns observed with other environmental carcinogens [30]. Although very short intervals, such as SCC diagnosed 6 months after tattooing, have been documented, these rapid onsets strongly suggest confounding, particularly the presence of pre-existing lesions masked by the tattoo [45, 46]. In such cases, tattoo-related trauma may simply reveal, modify, or accelerate the detection of an already evolving malignancy rather than represent a direct carcinogenic effect. Conversely, the longer latency intervals observed do not exclude a meaningful association; rather, our results suggest the possibility of a real association, potentially mediated by slow pigment degradation and cumulative biological effects.

This systematic review and meta-analysis highlight the need for cautious clinical and epidemiological interpretation. Although no consistent association was identified between tattoo exposure and cutaneous malignancy, the observed signal for hematologic cancers in sensitivity analysis warrants further evaluation in rigorously designed longitudinal studies. Uncertainty regarding temporality between tattoo exposure and cancer diagnosis raises the possibility of reverse causation, particularly in studies relying on retrospective exposure assessment. At present, the evidence supports continued clinical surveillance rather than modifications to current tattooing practices. Future research should prioritize large, prospective cohorts with standardized, high-resolution characterization of tattoo exposure, extended follow-up, and comprehensive control of key confounders to better define temporality, generalizability, and potential biological mechanisms.

## Limitations

Our results should be interpreted in light of several important limitations inherent to the available evidence. Key confounders were often unadjusted, and some attempted adjustments were not always feasible, making it difficult to isolate the effect of tattoo exposure from related lifestyle and environmental factors. Most studies also lack detailed baseline data on these confounders (e.g., family history and solar exposure), and study design (e.g., cohort and case–control). Tattoo exposure assessment is imprecise, with limited information on ink composition, inconsistent reporting of pigment colors or tattooed surface area, and frequent reliance on self-reported data, increasing the risk of exposure misclassification. Additionally, studies cannot establish temporality and, therefore, cannot infer causality. Inadequate assessment of pre-existing lesions, especially melanocytic lesions that may be masked by tattoos, further complicates interpretation. Finally, the predominance of studies from the Northern Hemisphere limits generalizability to regions with differing UV exposure, pigment regulations, and tattooing practices.

## Conclusion

In summary, this meta-analysis found no consistent association between tattoo exposure and cutaneous malignancies. While a signal for hematologic cancers emerged in sensitivity analyses, this finding is exploratory and does not alter the primary null result. Overall, the current evidence does not support a causal relationship; further well-designed prospective studies are warranted to clarify potential long-term risks.

### What is already known about this topic?

- Tattoo inks may contain substances classified as carcinogenic or probably carcinogenic.
- Pigment particles can migrate to lymphatic tissues and may induce chronic inflammatory responses.
- Previous systematic reviews have not demonstrated consistent evidence of an association between tattoo exposure and hematological malignancies.
- Epidemiological data regarding tattoo exposure and skin cancer remain limited and inconsistent.

### What does this study add?

- This updated meta-analysis synthesizes evidence from 140,841 participants across seven observational studies.
- No significant association was found between tattoo exposure and overall skin cancer.
- A significant association with hematological malignancies emerged only after sensitivity analysis.

The certainty of evidence ranged from low to moderate, highlighting methodological limitations in existing studies.

## Supplementary Information

Below is the link to the electronic supplementary material.

Supplementary file1 (DOCX 2523 KB)

## References

[1] Kluger N. Epidemiology of tattoos in industrialized countries. Curr Probl Dermatol. 2015;48:6–20. 10.1159/000369175. 25833619

[2] Renzoni A, Pirrera A, Novello F, Lepri A, Cammarata P, Tarantino C, et al. The tattooed population in Italy: a national survey on demography, characteristics and perception of health risks. Ann Ist Super Sanita. 2018;54:126–36. 10.4415/ANN_18_02_08. 29916417

[3] Kluger N, Seité S, Taieb C. The prevalence of tattooing and motivations in five major countries over the world. J Eur Acad Dermatol Venereol. 2019;33(12):e484–6. 10.1111/jdv.15808. 31310367

[4] Karagas MR, Wasson JH. A World Wide Web-based survey of nonmedical tattooing in the United States. J Am Acad Dermatol. 2012;66(1):e13–4. 10.1016/j.jaad.2010.11.038. PMC3478135 22177648

[5] Laumann AE, Derick AJ. Tattoos and body piercings in the United States: a national data set. J Am Acad Dermatol. 2006;55(3):413–21. 10.1016/j.jaad.2006.03.026. 16908345

[6] Mayers L, Chiffriller S. Sequential survey of body piercing and tattooing prevalence and medical complication incidence among college students. Arch Pediatr Adolesc Med. 2007;161(12):1219–20. 10.1001/archpedi.161.12.1219-b. 18056571

[7] Forte G, Petrucci F, Cristaudo A, Bocca B. Market survey on toxic metals contained in tattoo inks. Sci Total Environ. 2009;407(23):5997–6002. 10.1016/j.scitotenv.2009.08.034. 19766292

[8] McCarty RD, Trabert B, Kriebel D, Millar MM, Birmann BM, Grieshober L, et al. Tattoos and risk of hematologic cancer: a population-based case-control study in Utah. Cancer Med. 2024;13:e70260. 10.1002/cam4.70260. PMC11499570 39444249

[9] Kluger N, Koljonen V. Tattoos, inks, and cancer. Lancet Oncol. 2012;13(4):e161–8. 10.1016/S1470-2045(11)70340-0. 22469126

[10] Foerster M, Dufour L, Bäumler W, Schreiver I, Goldberg M, Zins M, et al. Development and validation of the epidemiological tattoo assessment tool to assess ink exposure and related factors in tattooed populations for medical research: cross-sectional validation study. JMIR Form Res. 2023;7:e42158. 10.2196/42158. PMC9878366 36630184

[11] Serup J, Severin BC, Hammershøy E. Tattoo ink products and pigments 2018–2019 upfront the new EU-REACH regulation: Danish golden benchmark study of 108 studios and 39,687 clients tattooed with inks from 109,720 ink bottles. Dermatology. 2025;241(3):259–71. 10.1159/000543455. PMC12324700 39799936

[12] Ćwieląg-Drabek M, Furman J, Gut-Pietrasz K. Heavy metal content in tattoo and permanent makeup inks and European standards: is there still a health risk? Toxics. 2025;13(11):934. 10.3390/toxics13110934. PMC12656104 41304486

[13] Moubadder L, McCullough LE, Flowers CR, Koff JL. Linking environmental exposures to molecular pathogenesis in Non-Hodgkin Lymphoma subtypes. Cancer Epidemiol Biomark Prev. 2020;29(10):1844–55. 10.1158/1055-9965.EPI-20-0228. PMC7541593 32727723

[14] Schreiver I, Hesse B, Seim C, Castillo-Michel H, Villanova J, Laux P, et al. Synchrotron-based ν-XRF mapping and μ-FTIR microscopy enable to look into the fate and effects of tattoo pigments in human skin. Sci Rep. 2017;7:11395. 10.1038/s41598-017-11721-z. PMC5595966 28900193

[15] Lehner K, Santarelli F, Vasold R, Penning R, Sidoroff A, König B, et al. Black tattoos entail substantial uptake of genotoxicpolycyclic aromatic hydrocarbons (PAH) in human skin and regional lymph nodes. PLoS One. 2014;9:e92787. 10.1371/journal.pone.0092787. PMC3966813 24670978

[16] Schreiver I, Hesse B, Seim C, Castillo-Michel H, Anklamm L, Villanova J, et al. Distribution of nickel and chromium containing particles from tattoo needle wear in humans and its possible impact on allergic reactions. Part Fibre Toxicol. 2019;16:33. 10.1186/s12989-019-0317-1. PMC6710876 31451117

[17] Sepehri M, Sejersen T, Qvortrup K, Lerche CM, Serup J. Tattoo pigments are observed in the Kupffer cells of the liver indicating blood-borne distribution of tattoo ink. Dermatology. 2017;233(1):86–93. 10.1159/000468149. 28486229

[18] Lehner K, Santarelli F, Penning R, Vasold R, Engel E, Maisch T, et al. The decrease of pigment concentration in red tattooed skin years after tattooing. J Eur Acad Dermatol Venereol. 2011;25:1340–5. 10.1111/j.1468-3083.2011.03987.x. 21349116

[19] Kaur RR, Kirby W, Maibach H. Cutaneous allergic reactions to tattoo ink. J Cosmet Dermatol. 2009;8(4):295–300. 10.1111/j.1473-2165.2009.00469.x. 19958434

[20] Kluger N. Cutaneous and systemic complications associated with tattooing. Presse Med. 2016;45(6 Pt 1):567–76. 10.1016/j.lpm.2016.02.016. 27160631

[21] Kalaora S, Nagler A, Wargo JA, Samuels Y. Mechanisms of immune activation and regulation: lessons from melanoma. Nat Rev Cancer. 2022;22(4):195–207. 10.1038/s41568-022-00442-9. 35105962

[22] McConnell T, Xu J, Freeman J, Zak I, Frain J. Investigating the potential association between tattoos and lymphoma: an exploratory systematic review and meta-analysis. EClinicalMedicine. 2025;89:103563. 10.1016/j.eclinm.2025.103563. PMC12547158 41140458

[23] Benandi K, Sieving D, Martin K, Wolf K. Malignant melanoma arising within tattoos as a risk factor for delayed diagnosis: a case report and systematic review. Dermatol Online J. 2025;31(3):10.5070/D331365358. 10.5070/D331365358. 40991465

[24] Lebhar J, Jacobs J, Rundle C, Kaplan SJ, Mosca PJ. Skin cancers arising within tattoos: a systematic review. JAAD Int. 2024;16:133–43. 10.1016/j.jdin.2024.03.015. PMC11217691 38957835

[25] Moher D, Liberati A, Tetzlaff J, Altman DG, PRISMA Group. Preferred reporting items for systematic reviews and meta-analyses: the PRISMA statement. PLoS Med. 2009;6(7):e1000097. 10.1371/journal.pmed.1000097. PMC2707599 19621072

[26] Cumpston M, Li T, Page MJ, Chandler J, Welch VA, Higgins JP, et al. Updated guidance for trusted systematic reviews: a new edition of the Cochrane Handbook for Systematic Reviews of Interventions. Cochrane Database Syst Rev. 2019;10:ED000142. 10.1002/14651858.ED000142. PMC10284251 31643080

[27] Brooke BS, Schwartz TA, Pawlik TM. MOOSE reporting guidelines for meta-analyses of observational studies. JAMA Surg. 2021;156(8):787–8. 10.1001/jamasurg.2021.0522. 33825847

[28] Higgins JPT, Morgan RL, Rooney AA, Taylor KW, Thayer KA, Silva RA, et al. A tool to assess risk of bias in non-randomized follow-up studies of exposure effects (ROBINS-E). Environ Int. 2024;186: 108602. 10.1016/j.envint.2024.108602. PMC11098530 38555664

[29] Clemmensen SB, Mengel-From J, Kaprio J, Frederiksen H, von Bornemann Hjelmborg J. Tattoo ink exposure is associated with lymphoma and skin cancers: a Danish study of twins. BMC Public Health. 2025;25(1):170. 10.1186/s12889-025-21413-3. PMC11736920 39819495

[30] Nielsen C, Jerkeman M, Jöud AS. Tattoos as a risk factor for malignant lymphoma: a population-based case–control study. EClinicalMedicine. 2024;72:102649. 10.1016/j.eclinm.2024.102649. PMC11141277 38827888

[31] Warner FM, Darvishian M, Boyle T, Brooks-Wilson AR, Connors JM, Lai AS, et al. Tattoos and hematologic malignancies in British Columbia. Canada Cancer Epidemiol Biomarkers Prev. 2020;29:2093–5. 10.1158/1055-9965.EPI-20-0515. 32699076

[32] Liljedahl ER, Engfeldt M, Nielsen K, Jöud A, Nielsen C. Tattoos and cutaneous squamous cell carcinoma: a population-based case–control study. Eur J Epidemiol. 2025;40(4):451–61. 10.1007/s10654-025-01230-z. PMC12145309 40278967

[33] McCarty RD, Trabert B, Collin LJ, Millar MM, Kriebel D, Grieshober L, et al. Tattooing and risk of melanoma: a population-based case-control study in Utah. J Natl Cancer Inst. 2025;117:2495–504. 10.1093/jnci/djaf235. PMC12404683 40839395

[34] Mo T, Zins M, Goldberg M, Ribet C, Kab S, Schreiver I, et al. Tattoos and risk of cutaneous melanoma and nonmelanoma skin cancer in France. J Natl Cancer Inst. 2026;118:476–84. 10.1093/jnci/djaf332. PMC13017047 41259064

[35] Paprottka FJ, Krezdorn N, Narwan M, Turk M, Sorg H, Noah EM, et al. Trendy tattoos-maybe a serious health risk? Aesthetic Plast Surg. 2018;42:310–21. 10.1007/s00266-017-1002-0. 29124377

[36] Fraga GR, Prossick TA. Tattoo-associated keratoacanthomas: a series of 8 patients with 11 keratoacanthomas. J Cutan Pathol. 2010;37(1):85–90. 10.1111/j.1600-0560.2009.01286.x. 19302572

[37] Joyce CW, Duff G, McKenna D, Regan PJ. Malignant melanoma arising in red tattoo ink. Arch Plast Surg. 2015;42(4):475–7. 10.5999/aps.2015.42.4.475. PMC4513057 26217569

[38] Barton DT, Karagas MR, Samie FH. Eruptive squamous cell carcinomas of the keratoacanthoma type arising in a cosmetic lip tattoo. Dermatol Surg. 2015;41(10):1190–3. 10.1097/DSS.0000000000000459. 26336055

[39] Ricci F, Paradisi A, Maier SA, et al. Melanoma and tattoos: a case report and review of the literature. Eur J Dermatol. 2018;28(1):50–5. 10.1684/ejd.2017.3184. 29171408

[40] Al-Gawahiri M, van der Bent SAS, Clahsen P, Vos LE. Single and multiple keratoacanthomas after a recent black tattoo in two patients. BMJ Case Rep. 2024;17:e260760. 10.1136/bcr-2024-260760. PMC11590445 40242860

[41] Cherkaoui El Baraka F, Kluger N, Ollivier I, Bourgoin R, Grossin M, Zeboulon C, et al. Melanoma within tattoos: two cases and a systematic literature review. Ann Dermatol Venereol. 2020;147:285–92. 10.1016/j.annder.2019.10.024. 31812363

[42] Monfrecola G, Fornaro L, Ascierto PA, et al. Could wide tattoo delay the early diagnosis of cutaneous melanoma? J Eur Acad Dermatol Venereol. 2023;37(9):e1117–8. 10.1111/jdv.19037. 36912734

[43] Nolan KA, Kling M, Birge M, Kling A, Fishman S, Phelps R. Melanoma arising in a tattoo: case report and review of the literature. Cutis. 2013;92(5):227–30. 10.3390/cancers17050767. 24343207

[44] Goldenberg G, Patel S, Patel MJ, Williford P, Sangueza O. Eruptive squamous cell carcinomas, keratoacanthoma type, arising in a multicolor tattoo. J Cutan Pathol. 2008;35(1):62–4. 10.1111/j.1600-0560.2007.00764.x. 18095997

[45] Kazlouskaya V, Junkins-Hopkins JM. Pseudoepitheliomatous hyperplasia in a red pigment tattoo: a separate entity or hypertrophic lichen planus-like reaction? J Clin Aesthet Dermatol. 2015;8(12):48–52. PMC4689511 26705448

[46] Pitarch G, Martínez-Menchón T, Martínez-Aparicio A, Sánchez-Carazo JL, Muñoz D, Fortea JM. Squamous cell carcinoma over tattoos. J Am Acad Dermatol. 2007;56(6):1072–3. 10.1016/j.jaad.2007.02.032. 17504728

