# Supplementary material for: Is tattooing associated with an increased risk of cancer? A systematic review and meta-analysis
Source: Clin Transl Oncol. 2026 Jun 6;28(11):5570–80. doi: 10.1007/s12094-026-04388-4 (PMC13694300; doi:10.1007/s12094-026-04388-4)
Supplement: Supplementary file 1 [file 12094_2026_4388_MOESM1_ESM.docx]

Supplementary Material

Is Tattooing Associated With an Increased Risk of Cancer? A Systematic Review and Meta-analysis

# Summary

# 1. Supplementary Figures

# 1.1 Meta-analyses and Subgroup Analyses

# Figure S1. Association between tattoo exposure of one session and skin cancer risk.

# Figure S2. Association between tattoo exposure of two or three sessions and skin cancer risk.

# Figure S3. Association between tattoo exposure of four or more sessions and skin cancer risk.

# Figure S4. Association between tattoo exposure and NHL.

# Figure S5. Association between tattoo exposure and Hodgkin lymphoma.

# Figure S6. Association between tattoo exposure and diffuse large B-cell lymphoma.

# Figure S7. Association between tattoo exposure and T-cell lymphoma.

# Figure S8. Association between tattoo exposure and follicular B-cell lymphoma.

# Figure S9. Association between tattoo exposure and other B-cell subtypes

# 1.2 Sensitivity Analyses: Leave-One-Out

# Figure S10. Leave-one-out analysis for skin cancer (overall).

# Figure S11. Leave-one-out analysis for lymphoma (overall).

# Figure S12. Leave-one-out analysis for non-Hodgkin lymphoma.

# Figure S13. Leave-one-out analysis for diffuse large B-cell lymphoma.

# 2. Supplementary Tables

# Table S1. PRISMA 2020 checklist

# Table S2. MOOSE checklist

Table S3. Risk of bias assessment of included studies using the ROBINS-E tool

Table S4. Detailed reasons for exclusion

Table S5. Skin Cancer (overall) data extraction

Table S6. Lymphoma (overall) data extraction

# 3. eMethods

# eMethods 1. Search Strategy

# eMethods 2. Data Extraction

eMethods 3. Quality Assessment: Risk of Bias Assessment Using ROBINS-E

# eMethods 4. Statistical Analysis

# 4. eResults

# Table S7. GRADE Assessment of Evidence Certainty

# Figure S1. Association between tattoo exposure of one session and skin cancer risk.

**Legend.** Odds ratio of association of skin cancer among patients with one tattoo session. The squares represent the weight of each study, with larger squares indicating studies with greater weight and more information. The black diamond below the studies shows the overall pooled effect derived from the included studies. The red bar below shows the prediction interval derived from the included studies. CI: Confidence Interval; OR: Odds Ratio; IV: Inverse Variance.

# Figure S2. Association between tattoo exposure of two or three sessions and skin cancer risk.

**Legend.** Odds ratio of association of skin cancer among patients with two or three tattoo sessions. The squares represent the weight of each study, with larger squares indicating studies with greater weight and more information. The black diamond below the studies shows the overall pooled effect derived from the included studies. The red bar below shows the prediction interval derived from the included studies. CI: Confidence Interval; OR: Odds Ratio; IV: Inverse Variance.

# Figure S3. Association between tattoo exposure of four or more sessions and skin cancer risk.

**Legend.** Odds ratio of association of skin cancer among patients with four or more tattoo sessions. The squares represent the weight of each study, with larger squares indicating studies with greater weight and more information. The black diamond below the studies shows the overall pooled effect derived from the included studies. The red bar below shows the prediction interval derived from the included studies. CI: Confidence Interval; OR: Odds Ratio; IV: Inverse Variance.

# Figure S4. Association between tattoo exposure and NHL.

**Legend.** Odds ratio of association of NHL among patients with tattoo. The squares represent the weight of each study, with larger squares indicating studies with greater weight and more information. The black diamond below the studies shows the overall pooled effect derived from the included studies. The red bar below shows the prediction interval derived from the included studies. CI: Confidence Interval; OR: Odds Ratio; IV: Inverse Variance.

# Figure S5. Association between tattoo exposure and Hodgkin lymphoma.

**Legend.** Odds ratio of association of HL among patients with tattoo. The squares represent the weight of each study, with larger squares indicating studies with greater weight and more information. The black diamond below the studies shows the overall pooled effect derived from the included studies. The red bar below shows the prediction interval derived from the included studies. CI: Confidence Interval; OR: Odds Ratio; IV: Inverse Variance.

# Figure S6. Association between tattoo exposure and diffuse large B-cell lymphoma.

**Legend.** Odds ratio of association of diffuse large B-cell lymphoma subtype among patients with tattoo. The squares represent the weight of each study, with larger squares indicating studies with greater weight and more information. The black diamond below the studies shows the overall pooled effect derived from the included studies. The red bar below shows the prediction interval derived from the included studies. CI: Confidence Interval; OR: Odds Ratio; IV: Inverse Variance.

# Figure S7. Association between tattoo exposure and T-cell lymphoma.

**Legend.** Odds ratio of association of T-cell lymphoma among patients with tattoo. The squares represent the weight of each study, with larger squares indicating studies with greater weight and more information. The black diamond below the studies shows the overall pooled effect derived from the included studies. The red bar below shows the prediction interval derived from the included studies. CI: Confidence Interval; OR: Odds Ratio; IV: Inverse Variance.

# Figure S8. Association between tattoo exposure and follicular B-cell lymphoma.

**Legend.** Odds ratio of association of follicular B-cell lymphoma among patients with tattoo. The squares represent the weight of each study, with larger squares indicating studies with greater weight and more information. The black diamond below the studies shows the overall pooled effect derived from the included studies. The red bar below shows the prediction interval derived from the included studies. CI: Confidence Interval; OR: Odds Ratio; IV: Inverse Variance.

# Figure S9. Association between tattoo exposure and other B-cell subtypes

**Legend.** Odds ratio of association of other B-cell subtypes among patients with tattoo. The squares represent the weight of each study, with larger squares indicating studies with greater weight and more information. The black diamond below the studies shows the overall pooled effect derived from the included studies. The red bar below shows the prediction interval derived from the included studies. CI: Confidence Interval; OR: Odds Ratio; IV: Inverse Variance.

# Figure S10. Leave-one-out analysis for skin cancer (overall).

**Legend.** Leave-one-out analysis of association of skin cancer (overall) among patients with tattoo. Each point represents the pooled effect size recalculated after omitting the corresponding study, with horizontal lines indicating 95% confidence intervals.

# Figure S11. Leave-one-out analysis for lymphoma (overall).

**Legend.** Leave-one-out analysis of association of lymphoma (overall) among patients with tattoo. Each point represents the pooled effect size recalculated after omitting the corresponding study, with horizontal lines indicating 95% confidence intervals.

# Figure S12. Leave-one-out analysis for non-Hodgkin lymphoma.

**Legend.** Leave-one-out analysis of association of NHL among patients with tattoo. Each point represents the pooled effect size recalculated after omitting the corresponding study, with horizontal lines indicating 95% confidence intervals.

# Figure S13. Leave-one-out analysis for diffuse large B-cell lymphoma.

**Legend.** Leave-one-out analysis of association of diffuse large B-cell lymphoma subtype among patients with tattoo. Each point represents the pooled effect size recalculated after omitting the corresponding study, with horizontal lines indicating 95% confidence intervals.

# Table S1. Checklist PRISMA

| **Section and Topic** | **Item #** | **Checklist item** | **Location where item is reported** |
| --- | --- | --- | --- |
| **TITLE** | | | |
| **Title** | 1 | Identify the report as a systematic review. | Page 1 |
| **ABSTRACT** | | | |
| **Abstract** | 2 | See the PRISMA 2020 for Abstracts checklist. | Page 2 |
| **INTRODUCTION** | | | |
| **Rationale** | 3 | Describe the rationale for the review in the context of existing knowledge. | Page 2 |
| **Objectives** | 4 | Provide an explicit statement of the objective(s) or question(s) the review addresses. | Page 3 |
| **METHODS** | | | |
| Eligibility criteria | 5 | Specify the inclusion and exclusion criteria for the review and how studies were grouped for the syntheses. | Page 3 |
| Information sources | 6 | Specify all databases, registers, websites, organisations, reference lists and other sources searched or consulted to identify studies. Specify the date when each source was last searched or consulted. | Page 3 |
| Search strategy | 7 | Present the full search strategies for all databases, registers and websites, including any filters and limits used. | Page 3 |
| Selection process | 8 | Specify the methods used to decide whether a study met the inclusion criteria of the review, including how many reviewers screened each record and each report retrieved, whether they worked independently, and if applicable, details of automation tools used in the process. | Page 3 |
| Data collection process | 9 | Specify the methods used to collect data from reports, including how many reviewers collected data from each report, whether they worked independently, any processes for obtaining or confirming data from study investigators, and if applicable, details of automation tools used in the process. | Page 3 |
| Data items | 10a | List and define all outcomes for which data were sought. Specify whether all results that were compatible with each outcome domain in each study were sought (e.g. for all measures, time points, analyses), and if not, the methods used to decide which results to collect. | Page 3 |
|  | 10b | List and define all other variables for which data were sought (e.g. participant and intervention characteristics, funding sources). Describe any assumptions made about any missing or unclear information. | Page 3 |
| Study risk of bias assessment | 11 | Specify the methods used to assess risk of bias in the included studies, including details of the tool(s) used, how many reviewers assessed each study and whether they worked independently, and if applicable, details of automation tools used in the process. | Page 3 |
| Effect measures | 12 | Specify for each outcome the effect measure(s) (e.g. risk ratio, mean difference) used in the synthesis or presentation of results. | Page 4 |
| Synthesis methods | 13a | Describe the processes used to decide which studies were eligible for each synthesis (e.g. tabulating the study intervention characteristics and comparing against the planned groups for each synthesis (item #5)). | Page 4 |
|  | 13b | Describe any methods required to prepare the data for presentation or synthesis, such as handling of missing summary statistics, or data conversions. | Page 4 |
|  | 13c | Describe any methods used to tabulate or visually display results of individual studies and syntheses. | Page 4 |
|  | 13d | Describe any methods used to synthesize results and provide a rationale for the choice(s). If meta-analysis was performed, describe the model(s), method(s) to identify the presence and extent of statistical heterogeneity, and software package(s) used. | Page 4 |
|  | 13e | Describe any methods used to explore possible causes of heterogeneity among study results (e.g. subgroup analysis, meta-regression). | Page 4 |
|  | 13f | Describe any sensitivity analyses conducted to assess robustness of the synthesized results. | Page 4 |
| Reporting bias assessment | 14 | Describe any methods used to assess risk of bias due to missing results in a synthesis (arising from reporting biases). | Page 4 |
| Certainty assessment | 15 | Describe any methods used to assess certainty (or confidence) in the body of evidence for an outcome. | Page 4 |
| **RESULTS** | | | |
| Study selection | 16a | Describe the results of the search and selection process, from the number of records identified in the search to the number of studies included in the review, ideally using a flow diagram. | Page 4 |
|  | 16b | Cite studies that might appear to meet the inclusion criteria, but which were excluded, and explain why they were excluded. | Page 4 |
| Study characteristics | 17 | Cite each included study and present its characteristics. | Page 5-6 |
| Risk of bias in studies | 18 | Present assessments of risk of bias for each included study. | Page 8 |
| Results of individual studies | 19 | For all outcomes, present, for each study: (a) summary statistics for each group (where appropriate) and (b) an effect estimate and its precision (e.g. confidence/credible interval), ideally using structured tables or plots. | Page 6-8 |
| Results of syntheses | 20a | For each synthesis, briefly summarise the characteristics and risk of bias among contributing studies. | Page 6-8 |
|  | 20b | Present results of all statistical syntheses conducted. If meta-analysis was done, present for each the summary estimate and its precision (e.g. confidence/credible interval) and measures of statistical heterogeneity. If comparing groups, describe the direction of the effect. | Page 6-8 |
|  | 20c | Present results of all investigations of possible causes of heterogeneity among study results. | Page 8 |
|  | 20d | Present results of all sensitivity analyses conducted to assess the robustness of the synthesized results. | Page 8 |
| Reporting biases | 21 | Present assessments of risk of bias due to missing results (arising from reporting biases) for each synthesis assessed. | Page 8 |
| Certainty of evidence | 22 | Present assessments of certainty (or confidence) in the body of evidence for each outcome assessed. | Page 8 |
| **DISCUSSION** | | | |
| Discussion | 23a | Provide a general interpretation of the results in the context of other evidence. | Page 8-9 |
|  | 23b | Discuss any limitations of the evidence included in the review. | Page 9-10 |
|  | 23c | Discuss any limitations of the review processes used. | Page 9-10 |
|  | 23d | Discuss implications of the results for practice, policy, and future research. | Page 11 |
| **OTHER INFORMATION** | | | |
| Registration and protocol | 24a | Provide registration information for the review, including register name and registration number, or state that the review was not registered. | Page 2 |
|  | 24b | Indicate where the review protocol can be accessed, or state that a protocol was not prepared. | Page 2 |
|  | 24c | Describe and explain any amendments to information provided at registration or in the protocol. | Page 2 |
| Support | 25 | Describe sources of financial or non-financial support for the review, and the role of the funders or sponsors in the review. | Page 1 |
| Competing interests | 26 | Declare any competing interests of review authors. | Page 1 |
| Availability of data, code and other materials | 27 | Report which of the following are publicly available and where they can be found: template data collection forms; data extracted from included studies; data used for all analyses; analytic code; any other materials used in the review. | Page 14-15 |

From: Page MJ, McKenzie JE, Bossuyt PM, Boutron I, Hoffmann TC, Mulrow CD, et al. The PRISMA 2020 statement: an updated guideline for reporting systematic reviews. BMJ 2021;372:n71. doi: 10.1136/bmj.n71. This work is licensed under CC BY 4.0. To view a copy of this license, visit <https://creativecommons.org/licenses/by/4.0/>

# Table S2. MOOSE checklist

| **Item No.** | **Recommendation** | **Brief description of how the criteria were handled in the meta-analysis** |
| --- | --- | --- |
| **Reporting of background should include** | | |
| 1 | Problem definition | No meta-analysis has comprehensively synthesized the available evidence to clarify the association between tattoo and skin cancer and hematological malignancies |
| 2 | Hypothesis statement | Tattoo ink is associated with an increased risk of developing cancer |
| 3 | Description of study outcome(s) | Description of study outcomes are detailed in the text. |
| 4 | Type of exposure or intervention used | We included original articles that reported the association of cancer in tattooed patients |
| 5 | Type of study designs used | Longitudinal |
| 6 | Study population | Individuals with cancer exposed to tattoo |
| **Reporting of search strategy should include** | | |
| 7 | Qualifications of searchers | The credentials of the investigators are indicated in the author list. |
| 8 | Search strategy, including time period included in the synthesis and key words | A multi-step literature search was conducted, which is detailed in the methods section, until January, 2026 |
| 9 | Effort to include all available studies, including contact with authors | All efforts were made to include unpublished and ongoing studies. |
| 10 | Databases and registries searched | A multi-step literature search was conducted, which is detailed in the methods section |
| 11 | Search software used, name and version, including special features used | We detailed the search software in “Methods Section”. |
| 12 | Use of hand searching | We hand-searched bibliographies of retrieved papers and published reviews for additional references. |
| 13 | List of citations located and those excluded, including justification | Details of the literature search process are outlined in the results section and in the PRISMA flowchart |
| 14 | Method of addressing articles published in languages other than English | We included articles in any language and used freely available software as needed. |
| 15 | Method of handling abstracts and unpublished studies | We looked for both abstracts and unpublished studies in the databases detailed above. |
| 16 | Description of any contact with authors | We planned to contact corresponding authors and authors from to request additional data when needed but this was not required. |
| **Reporting of methods should include** | | |
| 17 | Description of relevance or appropriateness of studies assembled for assessing the hypothesis to be tested | Our inclusion/ exclusion criteria, including the designs selected are appropriate to answer our research question. |
| 18 | Rationale for the selection and coding of data | Data extraction is in accordance with the population characteristics, study design, exposure, outcome, and possible effect of confounders. |
| 19 | Documentation of how data were classified and coded | Data were extracted according to a predefined protocol and coded into standardized categories. A second reviewer verified the coding for accuracy. |
| 20 | Assessment of confounding | Meta-regressions were carried out as detailed in the main text. |
| 21 | Assessment of study quality, including blinding of quality assessors, stratification or regression on possible predictors of study results | We used the ROBINS-E to assess the quality of the study. |
| 22 | Assessment of heterogeneity | Heterogeneity was assessed with the I2 index.. |
| 23 | Description of statistical methods | Statistical methods are detailed in the methods section so they can be replicated. |
| 24 | Provision of appropriate tables and graphics | Both tables and graphics are provided in the main text and the supplementary materials. |
| **Reporting of results should include** | | |
| 25 | Graphic summarizing individual study estimates and overall estimate | Graphs are included in the main text and supplementary, summarizing estimates. |
| 26 | Table giving descriptive information for each study included | Our Table in the main text provides descriptive information for each study included. |
| 27 | Results of sensitivity testing | Sensitivity analysis are reported as detailed in the methods section. |
| 28 | Indication of statistical uncertainty of findings | All our meta-analysis include 95% CIs. |

| **Item No** | **Recommendation** | **Brief description of how the criteria were handled in the meta-analysis** |
| --- | --- | --- |
| Reporting of discussion should include | | |
| 29 | Quantitative assessment of bias | We evaluated publication bias as explained in our methods. |
| 30 | Justification for exclusion | Our exclusion criteria as specified in the text aim to obtain the evidence that helps us answer our research question. |
| 31 | Assessment of quality of included studies | An assessment of the quality of included studies is provided in the main text and supplementary text. |
| **Reporting of conclusions should include** | | |
| 32 | Consideration of alternative explanations for observed results | We have discussed alternative explanations for our findings in the discussion of the text. |
| 33 | Generalization of the conclusions | We have addressed the generalization of the conclusions in the discussion of the manuscript. |
| 34 | Guidelines for future research | We have suggested guidelines for future research in the discussion of the manuscript. |
| 35 | Disclosure of funding source | No separate funding was necessary for the undertaking of this meta-analysis. Conflicts of interest for all the coauthors and funding sources were detailed. |

***From*:** Stroup DF, Berlin JA, Morton SC, et al, for the Meta-analysis Of Observational Studies in Epidemiology (MOOSE) Group. Meta-analysis of Observational Studies in Epidemiology. A Proposal for Reporting. *JAMA*. 2000;283(15):2008-2012. doi: 10.1001/jama.283.15.2008.

# **Table S**3**.** Risk of Bias Assessment Using ROBINS-E

| Study | D1 | D2 | D3 | D4 | D5 | D6 | D7 | Overall |
| --- | --- | --- | --- | --- | --- | --- | --- | --- |
| Liljedahl, 2025 | Some concerns | Low | Some concerns | Low | Some concerns | Some concerns | Low | Some concerns |
| McCarty, 2025 | High¹ | Low | Some concerns | Low | Some concerns | Low | Low | High |
| Clemmensen, 2025 | High¹ | Low | Some concerns | Low | Some concerns | Low | Low | High |
| Warner, 2020 | Some concerns | Low | Some concerns | Low | Low | Low | Low | Some concerns |
| Nielsen, 2024 | Some concerns | Some concerns | Low | Low | Low | Some concerns | Some concerns | Some concerns |
| McCarty, 2024 | High¹ | Low | Some concerns | Low | Some concerns | Low | Low | High |
| Mo, 2025 | High¹ | Some concerns | Some concerns | Some concerns | Low | Low | Low | High |

This table summarizes the domain-level and overall risk of bias assessment for the included observational studies, conducted with the Risk Of Bias in Non-randomized Studies of Exposure (ROBINS-E) tool.

ROBINS-E evaluates seven domains: D1: Bias due to confounding; D2: Bias in selection of participants into the study; D3: Bias in classification of exposures; D4: Bias due to post-exposure interventions; D5: Bias due to missing data; D6: Bias in measurement of outcomes; D7: Bias in selection of the reported result.

¹ High bias level due: Lack of information on UV exposure, or any other major confounder for skin cancer; Lack of information on family history

# Table S4. Detailed reasons for exclusion

| **Study** | **Journal** | **Title** | **DOI** | **Reason for exclusion** |
| --- | --- | --- | --- | --- |
| Theune LE et al., 2025 | PLoS One | Characteristics of a tattooed population and a possible role of tattoos as a risk factor for chronic diseases: Results from the LIFE-Adult-Study | 10.1371/journal.pone.0319229 | Cross-sectional population-based study evaluating tattoo prevalence and self-reported chronic diseases; no specific assessment of hematological malignancies, no incident cancer outcomes, and no effect estimates (OR/RR/HR) for cancer risk, precluding inclusion in quantitative synthesis. |
| Karregat, JJJP et al. 2025 | *Dermatology* | Incidence of Tattoo-Associated Melanoma in the Netherlands (1991-2023): A Nationwide Registry Study | 10.1159/000549503 | Missing Number of controls without tattoos, Number of controls with tattoos, Number of melanoma cases without tattoos, Tattoo prevalence in the source population. The registry only captures melanomas that occurred on tattooed skin |
| Barton DT et al. 2020 | Epidemiology | Cosmetic Tattooing and Early Onset Basal Cell Carcinoma: A Population-based Case-Control Study from New Hampshire | 10.1097/EDE.0000000000001179 | Case–control study restricted to basal cell carcinoma, This outcome was not evaluated in any other included study, and some studies excluded this type, preventing clinical and methodological comparability. Consequently, inclusion would preclude pooled quantitative synthesis, and introduce undue heterogeneity without contributing to the primary research question. |
| Corona R et al. 2001 | Arch Dermatol | Risk factors for basal cell carcinoma in a Mediterranean population: role of recreational sun exposure early in life | 10.1001/archderm.137.9.1162 | Without tattoo as a variable |

# Table S5. Skin Cancer (overall) data extraction

| Clemmensen et al. 2025 | | | Liljedahl et al. 2025 | | |
| --- | --- | --- | --- | --- | --- |
| Outcome/Exposure | Tattoo | No Tattoo | Outcome/Exposure | Tattoo | No Tattoo |
| Cancer | 30 | 89 | Cancer | 213 | 1216 |
| No Cancer | 25 | 79 | No Cancer | 418 | 2191 |

# Table S6. Lymphoma (overall) data extraction

| Clemmensen et al. 2025 | | | McCarty et al. 2024 | | | McCarty et al. 2024 | | |
| --- | --- | --- | --- | --- | --- | --- | --- | --- |
| Outcome/Exposure | Tattoo | No Tattoo | Outcome/Exposure | Tattoo | No Tattoo | Outcome/Exposure | Tattoo | No Tattoo |
| Cancer | 10 | 22 | Cancer | 79 | 562 | Cancer | 79 | 562 |
| No Cancer | 11 | 23 | No Cancer | 961 | 5449 | No Cancer | 961 | 5449 |

# eMethods 1. Search Strategy

PUBMED: 1,317

1."Tattooing"[Mesh] OR "Tattoos"[Mesh] OR tattoo*[tiab] OR tattooing[tiab] OR "tattoo ink"[tiab]

2. "Skin Neoplasms"[Mesh] OR "Melanoma"[Mesh] OR "Lymphoma"[Mesh] OR "Non-Hodgkin Lymphoma"[Mesh] OR "Multiple Myeloma"[Mesh] OR "Neoplasms"[Mesh] OR cutaneous neoplasm*[tiab] OR skin cancer*[tiab] OR melanoma*[tiab] OR carcinoma*[tiab] OR lymphoma*[tiab] OR "non-Hodgkin lymphoma"[tiab] OR myeloma*[tiab] OR "cancer risk"[tiab] OR carcinogen*[tiab] OR "lymphatic cancer*"[tiab]

3. 1 AND 2

EMBASE 1,333

1. ('tattooing'/exp OR 'tattoo'/exp OR tattoo*:ti,ab OR tattooing:ti,ab OR 'tattoo ink':ti,ab)

2. ('skin tumor'/exp OR 'melanoma'/exp OR 'lymphoma'/exp OR 'non hodgkin lymphoma'/exp OR 'multiple myeloma'/exp OR 'neoplasm'/exp OR cutaneous neoplasm*:ti,ab OR skin cancer*:ti,ab OR melanoma*:ti,ab OR carcinoma*:ti,ab OR lymphoma*:ti,ab OR 'non hodgkin lymphoma':ti,ab OR myeloma*:ti,ab OR 'cancer risk':ti,ab OR carcinogen*:ti,ab OR 'lymphatic cancer*':ti,ab)

3. 1 AND 2

Cochrane: 80

1. (tattoo* OR tattooing OR "tattoo ink")

2. (neoplasm* OR melanoma OR lymphoma OR carcinoma OR myeloma OR "skin cancer*" OR "non-Hodgkin lymphoma")

3. 1 AND 2

Titles and abstracts were screened independently and in duplicate by two reviewers (G.C.N.T. amd G.M.). Full texts of potentially eligible studies were subsequently assessed independently by the same reviewers. Discrepancies were resolved by discussion with two additional reviewers (A.U.K. and P.S.) when necessary. No automation tools or artificial intelligence–assisted software were used during title/abstract screening or full-text assessment. No language restrictions were applied, studies published in languages other than English were screened and assessed using reviewer language proficiency and, when required, translation to ensure accurate eligibility evaluation. Additional studies were identified through manual backward and forward reference searching, including screening reference lists of included articles and relevant reviews, as well as articles citing the included studies. When information required to assess eligibility was missing or unclear, the corresponding author and, when necessary, the first or last author were contacted to obtain clarification.

# eMethods 2. Data Extraction

a) “The prevalence of tattooing was 22% of 79 among HL cases, 11% of 562 among NHL cases, and 15% among controls.” (3.1 | Lymphoid Neoplasms, McCarty 2024)
b) Crude odds ratios and 95% confidence intervals were calculated from 2×2 contingency tables using the log odds ratio and its standard error - when not available - in accordance with standard Cochrane methodological guidance.
c) Tattoo exposure status was defined dichotomously as ever tattooed versus never tattooed. Lymphoma outcomes were extracted according to the most granular subtype classification available in each study (e.g., Hodgkin lymphoma, non-Hodgkin lymphoma, diffuse large B-cell lymphoma), based on cancer registry data, histological confirmation, or standardized diagnostic criteria. When multiple subtype-specific effect estimates were reported within a single study, each estimate was extracted separately.

In addition, overall lymphoma outcomes (all subtypes combined) were extracted as reported in the main descriptive tables (e.g., Table 1) of the original studies. The same extraction approach was applied to skin cancer outcomes, both overall and by subtype when available.

d) All data extractions were independently reviewed by two investigators, with discrepancies resolved by consensus.
e) For outcomes not presented in Tables S5–S6, odds ratios were reported directly in the original studies.

# eMethods 3. Quality Assessment: Risk of Bias Assessment Using ROBINS-E

The methodological quality and risk of bias of included observational studies were independently assessed by two reviewers using the Risk Of Bias In Non-randomized Studies of Exposure (ROBINS-E) tool. This instrument evaluates seven domains:

**1.** Bias due to confounding (adequacy of controlling for age, sex, comorbidities, and UV).

**2.** Bias in selection of participants into the study (representativeness and exclusion criteria).

Bias in classification of exposures (Cases interviewed by trained staff vs self-report vs Questionnaire ).

**3.** Bias in classification of exposure, evaluating the method of tattoo exposure ascertainment (e.g., interviewer-administered assessment versus self-report or questionnaire-based data)

**4.** Bias due to post-exposure interventions, considering whether factors occurring after tattoo exposure but prior to outcome assessment could influence the observed associations.

**5.** Bias due to missing data, including completeness of exposure, covariate, and outcome information, and the handling of missingness and attrition.

**6.** Bias in measurement of outcomes, assessing the validity and reliability of outcome ascertainment (e.g., cancer registry data, histological confirmation, or standardized diagnostic criteria).

**7.** Bias in selection of the reported result, examining selective reporting of outcomes, analyses, or exposure contrasts.

Each domain, and the overall risk of bias, was rated as low risk, moderate risk, serious risk, critical risk, or no information, following ROBINS-E guidance. Results of the assessments were synthesized and visualized using the ROBVIS web tool, producing traffic-light plots and summary figures (Supplementary Table S3, Figures S1–S2).

Discrepancies between reviewers were resolved by consensus; when disagreement persisted, a third reviewer (E.S.F.) adjudicated. Inter-rater agreement was calculated prior to consensus.

# eMethods 4. Statistical Analysis

a) All statistical analyses were conducted using R software (R Foundation for Statistical Computing), primarily with the meta package, with supplementary functions from metafor for validation and sensitivity procedures.
b) Effect estimates were analyzed on the logarithmic scale, with standard errors derived from published confidence intervals using established methods. Pooled estimates were subsequently back-transformed for presentation.
c) Random-effects meta-analyses were performed throughout
d) Between-study variance (τ²) was estimated primarily using the restricted maximum likelihood (REML) method. In selected sensitivity analyses, the DerSimonian–Laird (DL) estimator was applied to assess robustness of results.
e) Separate meta-analyses were conducted according to:

Cancer outcome (skin cancer and malignant lymphoma),

Tattoo exposure intensity or frequency categories (e.g., 1 session, 2–3 sessions, ≥4 sessions),

Lymphoma subtypes, including overall lymphoma, non-Hodgkin lymphoma, Hodgkin lymphoma, diffuse large B-cell lymphoma, follicular lymphoma, T-cell lymphoma, and other B-cell lymphomas.

f) To assess the influence of individual studies on pooled estimates, prespecified leave-one-out sensitivity analyses were conducted using the metainf() function from the meta package when moderate or greater heterogeneity was observed (I² > 30%). Each study was sequentially excluded and pooled random-effects estimates and heterogeneity metrics were recalculated.
g) Leave-one-out analyses were additionally conducted in selected comparisons despite low statistical heterogeneity when visual inspection of forest plots or substantive methodological considerations (e.g., marked population heterogeneity or differences in exposure definition) suggested that individual studies might disproportionately influence the pooled estimate.

# Table S7. GRADE Assessment of Evidence Certainty

| **Certainty assessment** | | | | | | | **Effect size** | | **Certainty / Importance** | |
| --- | --- | --- | --- | --- | --- | --- | --- | --- | --- | --- |
| **Outcome** | **No. of studies** | **Study design** | **Risk of bias** | **Inconsistency** | **Indirectness** | **Imprecision** | **Other considerations** | **OR(95% CI)** | **Certainty** | **Importance** |
| Skin cancer (overall) | 4 | Observational | Seriousᵃ | Seriousᵇ | Not serious | Seriousᶜ | None | 0.92; 95% CI, 0.83–1.04 | ⨁⨁◯◯ Low | CRITICAL |
| One tattoo session | 2 | Observational | Seriousᵃ | Seriousᵇ | Not serious | Very serious³ | None | 1.08; 95% CI 0.55–2.12 | ⨁⨁◯◯ Low | IMPORTANT |
| Two or three tattoo sessions | 2 | Observational | Seriousᵃ | Seriousᵇ | Not serious | Very serious³ | None | 0.90; 95% CI 0.62–1.32 | ⨁◯◯◯ Very low | IMPORTANT |
| Four or more sessions | 2 | Observational | Seriousᵃ | Seriousᵇ | Not serious | Very serious³ | None | 0.70; 95% CI 0.29-1.70 | ⨁◯◯◯ Very low | IMPORTANT |
| Hematological malignancies | 4 | Observational | Seriousᵃ | Seriousᵇ | Not serious | Not serious | Raw OR was calculated based on the baseline characteristics reported in the included studies | 1.02; 95% CI 0.78–1.32 | ⨁⨁⨁◯ Moderate | CRITICAL |
| NHL overall | 3 | Observational | Seriousᵃ | Not serious | Not serious | Seriousᶜ | None | 1.00; 95% CI 0.73–1.36 | ⨁⨁◯◯ Low | CRITICAL |
| HL | 2 | Observational | Seriousᵃ | Not serious | Not serious | Seriousᶜ | None | 1.19; 95% CI 0.59–2.40 | ⨁⨁◯◯ Low | CRITICAL |
| Other B-cell lymphomas | 3 | Observational | Seriousᵃ | Not serious | Not serious | Seriousᶜ | None | 1.14 (0.83–1.55) | ⨁⨁◯◯ Low | IMPORTANT |
| T-cell lymphomas | 2 | Observational | Seriousᵃ | Not serious | Not serious | Seriousᶜ | None | 1.07 (0.61–1.88) | ⨁⨁◯◯ Low | IMPORTANT |
| DLBCL | 3 | Observational | Seriousᵃ | Seriousᵇ | Not serious | Seriousᶜ | None | 0.90 (0.57–1.44) | ⨁⨁◯◯ Low | IMPORTANT |
| Follicular B-cell lymphoma | 3 | Observational | Seriousᵃ | Not serious | Not serious | Seriousᶜ | None | 0.99 (0.72–1.36) | ⨁⨁◯◯ Low | IMPORTANT |

**Abbreviations:** CI: Confidence Interval; CRITICAL: Outcome rated as critical for decision-making (GRADE domain); GRADE: Grading of Recommendations Assessment, Development and Evaluation; SD: Standard Deviation.

GRADE-Related Symbols: ⨁⨁⨁⨁: high certainty of evidence; ⨁⨁⨁◯: Moderate certainty of evidence; ⨁⨁◯◯: Low certainty of evidence; ⨁◯◯◯: Very Low certainty of evidence.

ᵃ Downgraded one level for risk of bias: observational design with residual confounding.
ᵇ Downgraded one level for inconsistency: I² >50%.
ᶜ Downgraded one level for imprecision: wide confidence intervals crossing the line of no effect; very serious imprecision for four or more sessions due to extremely wide CI and small number of events.
